# miR‐196b‐5p inhibits proliferation of Wharton's jelly umbilical cord stem cells

**DOI:** 10.1002/2211-5463.13043

**Published:** 2020-12-08

**Authors:** Xiao Han, Haoqing Yang, Huina Liu, Chen Zhang, Yangyang Cao, Zhipeng Fan, Ruitang Shi

**Affiliations:** ^1^ Beijing Key Laboratory of Tooth Regeneration and Function Reconstruction Beijing Stomatological Hospital School of Stomatology Capital Medical University Beijing China; ^2^ Department of Endodontics Beijing Stomatological Hospital School of Stomatology Capital Medical University Beijing China

**Keywords:** cell cycle, cell proliferation, miR‐196‐5p, Wharton's jelly umbilical cord stem cells

## Abstract

Human umbilical cord mesenchymal stem cells can be obtained from different parts of the umbilical cord, including Wharton's jelly. Transplantation of Wharton's jelly umbilical cord stem cells (WJCMSCs) is a promising strategy for the treatment of various diseases. However, the molecular mechanisms underlying the proliferation of WJCMSCs are incompletely understood. Here, we report that overexpression of miR‐196b‐5p in WJCMSCs suppresses proliferation and arrests the cell cycle in G0/G1 phase, whereas knockdown of miR‐196b‐5p promotes WJCMSC proliferation and cell‐cycle progression. Moreover, miR‐196b‐5p overexpression resulted in decreased levels of Cyclin A, Cyclin D, Cyclin E and cyclin‐dependent kinases 2 and increased levels of p15^INK4b^, whereas miR‐196b‐5p knockdown had the opposite effects. In conclusion, our data suggests that miR‐196b‐5p inhibits WJCMSC proliferation by enhancing G0/G1‐phase arrest.

## Graphical abstract

We show here that overexpression of miR‐196b‐5p suppresses proliferation of Wharton's jelly umbilical cord stem cells (WJCMSCs) by arresting the cell cycle in G0/G1 phase through decreasing levels of Cyclin A, Cyclin D, Cyclin E and cyclin‐dependent kinases 2 and increasing levels of p15^INK4b^.

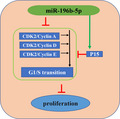

**Abbreviations**
BMMSC: bone marrow mesenchymal stem cell
CDK: cyclin‐dependent kinase
CFSE (CFDA‐SE): 5,6‐carboxyfluorescein diacetate, succinimidyl ester
GAPDH: glyceraldehyde‐3 phosphate dehydrogenase
iPSC: induced pluripotent stem cell
MSC: mesenchymal stem cell
qRT‐PCR: quantitative RT‐PCR
SA‐β‐gal: senescence‐associated β‐galactosidase
SWATH‐MS: sequential window acquisition of all theoretical mass spectra
WJCMSC: Wharton's jelly umbilical cord stem cell

Mesenchymal stem cells (MSCs) have a wide range of applications in regenerative medicine and immunotherapy because of their good ability of proliferation, multidirectional differentiation and immunoregulation [1, 2]. With the isolation and identification of bone marrow MSCs (BMMSCs), embryonic stem cells and induced pluripotent stem cells (iPSCs), these stem cells have become an important part of regeneration therapy [3, 4, 5]. However, the low proportion of BMMSCs in bone marrow, the ethical issues of embryonic stem cells extraction from embryos and the tumorigenicity of iPSCs limit their clinical application [6, 7, 8]. Therefore, it is imperative to find a useful source of stem cells.

Human umbilical cord MSCs can be obtained from different parts of the umbilical cord, including cord lining, perivascular region and Wharton’s jelly [9]. Wharton’s jelly umbilical cord stem cells (WJCMSCs) were used by most studies for their more prominent biological characteristics [10, 11]. First, the collection of WJCMSCs process is noninvasive and can prevent the risk for infection. In terms of the biological properties, WJCMSCs have higher proliferation potential than other MSCs. Furthermore, WJCMSCs have higher differentiation ability and faster self‐renewal ability than BMMSCs because of their unique gene expression profile, producing almost no teratomas and maintaining at an earlier embryonic stage [12, 13]. Moreover, some studies revealed that WJCMSCs are a good candidate for allogeneic transplantation because of their lower immunogenicity than BMMSCs [14]. The immunological characteristics of WJCMSCs also suggest that they may be a choice to treat autoimmune diseases such as type 1 diabetes [15]. Due to these advantages, WJCMSCs are the ideal source of stem cell for regenerative medicine and immunotherapy and have broad application prospects. The growth of WJCMSCs was influenced by the culture *in vitro*. When expanded to passage 10, the growth rate of WJCMSCs was slower than those at passage 5 [16]. Cell proliferation is important for cell therapy and tissue engineering because sufficient cell numbers are the prerequisite to ensure their regenerative and therapeutic effect. However, the molecular mechanisms of WJCMSC proliferation are vague. Therefore, it is of great significance to explore the molecular mechanisms of WJCMSC proliferation for better application of WJCMSCs in the future.

miRNAs are kinds of noncoding single‐stranded RNA molecules, which play a significant role in cell proliferation, cell apoptosis, cell cycle and differentiation by inducing target mRNA cleavage or translational inhibition [17, 18, 19, 20]. Previously, our miRNA array data showed that some miRNAs, including miR‐196b‐5p, were differentially expressed in WJCMSCs, adipose‐derived stem cells (ADSCs), BMMSCs, stem cells from apical papilla (SCAPs), dental pulp stem cells (DPSCs) and periodontal ligament stem cells (PDLSCs), which was confirmed by real‐time quantitative RT‐PCR (qRT‐PCR) (Fig. S1). These differentially expressed miRNAs may be key regulators controlling the proliferation and differentiation of these stem cells and warrant further study. miR‐196b gene is located in highly evolutionarily conserved regions of human chromosome 7 between *HOXA9* and *HOXA10* genes [21]. miR‐196b can regulate cell proliferation, invasion and migration in many tumors [22, 23]. miR‐196 might be involved in the process of chondrogenic differentiation of human iPSCs and was significantly up‐regulated in chondrocytes derived from human iPSCs [24]. Recently, it was revealed that miR‐196b inhibited human leukemia stem cell growth by targeting *Cdkn1b* [25]. However, the function of miR‐196b‐5p in WJCMSC proliferation is unclear.

In this study, the physiological function of miR‐196b‐5p on WJCMSC proliferation was investigated. We confirmed that miR‐196b‐5p suppressed cell proliferation and blocked G0/G1 phase of WJCMSCs, indicating that miR‐196b‐5p can be served as a potential target and help for clinic application of WJCMSCs in the future.

## Materials and methods

### Cell culture

The research involving human stem cells in this study complied with the International Society for Stem Cell Research ‘Guidelines for the Conduct of Human Embryonic Stem Cell Research’. Human WJCMSCs were purchased from ScienCell Research Laboratories (Carlsbad, CA, USA). Cells were cultured as shown previously [26]. Cells at generations 3–5 were used in the subsequent experiments.

### Induction of senescence and senescence‐associated β‐galactosidase staining

To induce premature senescence, we treated WJCMSCs with H_2_O_2_ (100 mm) for 4 h, washed with PBS and continued to incubate for 24 h. A senescence‐associated β‐galactosidase (SA‐β‐gal) staining kit (Cell Senescence Testing Kit; GenMed Scientifics Inc., Shanghai, China) was used following the manufacturer’s protocol. In brief, cells were washed and fixed with 1× Fixative Solution for 10 min at room temperature. Then the cells were incubated at 37 °C with β‐galactosidase staining solution (pH 6.0) for 24 h. The number of SA‐β‐gal‐positive cells was selected in 10 randomly chosen fields, and the percentage of positive cells was calculated from three independent experiments.

### Synthesis of miRNA and construction

The lentivirus miR‐196b‐5p mimic, miR‐196b‐5p inhibitor and negative control (Consh) were obtained from GenePharma (Suzhou, China). Virus transfection was performed as described previously [27]. The sequences are listed in Table 1.

**Table 1:** Sequences used in the study. F, forward; R, reverse.

Gene symbol	Sequence (5′–3′)
miR‐196b‐5p mimic	TAGGTAGTTTCCTGTTGTTGGG
miR‐196b‐5p NC mimic	GTTVTCCGAACGTGTCACGT
miR‐196b‐5p inhibitor	CCCAACAACAGGAAACTACCTA
miR‐196b‐5p NC inhibitor	CAGUACUUUUGUGUAGUACAA
miR‐196b‐5p	F: GCGTAGGTAGTTTCCTG
miR‐196b‐5p	R: GAGCAGGCTGGAGAA
U6	F: GCTTCGGCAGCACATATACT
U6	R: GAGCAGGCTGGAGAA
PTGS2	F: ATGCTGACTATGGCTACAAAAGC
PTGS2	R: TCGGGCAATCATCAGGCAC
PON2	F: GTTGGACCGGCATTTCTAT
PON2	R: CATTTGCCCAGTGTAAGTTCAAG
METTL3	F: AGATGGGTAGAAAGCCTCCT
METTL3	R: TGGTCAGCATAGGTTACAAGAGT
GAPDH	F: ACAACTTTGGTATCGTGGAAGG
GAPDH	R: GCCATCACGCCACAGTTTC

### RNA isolation and real‐time qRT‐PCR

Total RNA was extracted from transfected WJCMSCs by TRIzol (Invitrogen, Carlsbad, CA, USA). The levels of miRNA or mRNA in WJCMSCs were detected by using a Hairpin‐it™ microRNA and U6 snRNA Normalization RT‐PCR Quantitation Kit (GenePharma) or QuantiTect SYBR Green PCR kit (Qiagen, Hilden, Germany). U6 and *GAPDH* (*glyceraldehyde‐3 phosphate dehydrogenase*) were used to normalize miRNA or mRNA levels. The primer sequences are listed in Table 1.

### 5,6‐Carboxyfluorescein diacetate, succinimidyl ester assay

Following the CellTrace™ 5,6‐carboxyfluorescein diacetate, succinimidyl ester (CFSE) Cell Proliferation Kit protocol (Invitrogen), CFSE assay was determined as shown previously [27]. In brief, the suspension of cells was labeled with CFSE and inoculated into a six‐well plate at 1.0 × 10^5^ cells per plate for 72 h. The proliferation cells were fixed with formaldehyde and analyzed by flow cytometry (FACSCalibur; BD Biosciences, New Jersey, NJ, USA). Calculation of proliferation index was carried on by ModFit LT (Verity Software House, Topsham, ME, USA).

### Cell‐cycle assay

The transfected WJCMSCs were collected and fixed with cold 70% alcohol at 4 °C overnight, washed and resuspended with PBS. According to the operating protocol, the cells were added with 100 µg·mL^−1^ RNase A at 37 °C for 30 min and finally 100 µg·mL^−1^ Propidium (PI) (Sigma‐Aldrich, St. Louis, MO, USA) stained for 20 min away from light at 4 °C. DNA content was evaluated by ModFit LT. The proliferation index was calculated as PI = (S + G2/M)/(G0/G1 + S + G2/M).

### Western blot

Total proteins were resolved from transfected WJCMSCs, and SDS‐polyacrylamide gel tests were performed as shown previously [28]. The primary antibodies in this study were Cyclin A (Cat. No. SAB4503499; Sigma‐Aldrich), Cyclin D (Cat. No. 05‐152; Merck Millipore, Darmstadt, Germany), Cyclin E (Cat. No. 05‐363; Merck Millipore), cyclin‐dependent kinase 2 (CDK2; Cat. No. 05‐163; Merck Millipore), CDK4 (Cat. No. MAB8879; Merck Millipore), p15^INK4B^ (Cat. No. 4822; Cell Signaling Technology, Boston, MA, USA) and GAPDH (Cat. No. G8795; Sigma‐Aldrich). imagej 1.52 V (National Institutes of Health, Bethesda, MD, USA) was used to quantify the bands related to GAPDH expression and to normalize the total protein loaded in each lane.

### Statistical analysis

Statistical calculations were implemented using spss 10.0 statistical software (SPSS Inc, Chicago, IL, USA). Statistical significance was analyzed by Student’s *t*‐test or one‐way ANOVA; *P* ≤ 0.05 was regarded as statistically significant.

## Results

### The expression of miR‐196b‐5p is increased in senescent cells

**Fig. 1:**
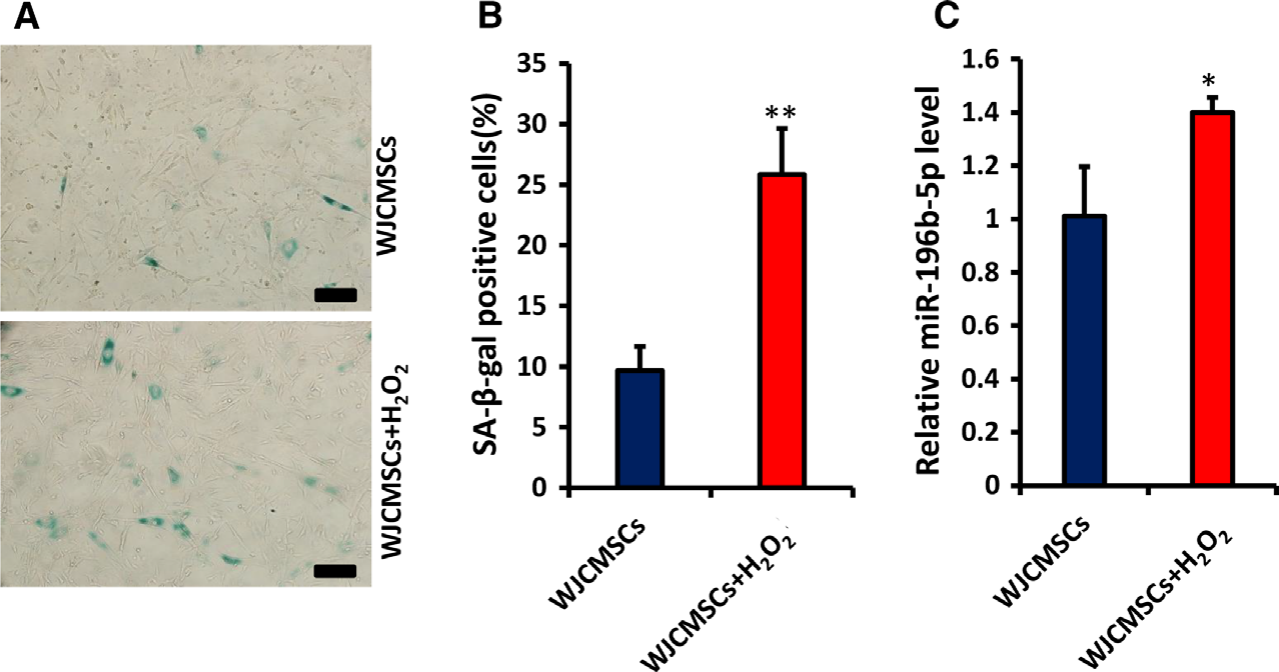
The expression of miR‐196b‐5p in senescent cells. (A, B) Senescence‐associated β‐galactosidase (SA‐β‐gal) staining (blue) and quantitative analysis of senescent WJCMSCs induced with H_2_O_2_. Scale bar: 100 μm. (C) Real‐time RT‐PCR showed the expression of miR‐196b‐5p increased in senescent WJCMSCs. U6 was used as an internal control for miR‐196b‐5p. Student’s *t*‐test was used to analyze the statistical significance. All error bars signify standard deviations (*n* = 3). *P ≤ 0.05, **P ≤ 0.01.

Senescence was induced with H_2_O_2_ in WJCMSCs as previously described to measure the effect of aging on the expression of miR‐196b‐5p [29]. SA‐β‐gal staining and quantitative analysis results showed that the percentage of SA‐β‐gal^+^ cells in the WJCMSCs + H_2_O_2_ group increased significantly compared with the control group (Fig. 1A,B). qRT‐PCR was used to measure the expression of miR‐196b‐5p in senescent cells, and the results indicated that the level of miR‐196b‐5p in the WJCMSCs + H_2_O_2_ group was significantly increased compared with the WJCMSCs group (Fig. 1C).

### Overexpression of miR‐196b‐5p suppresses cell proliferation and arrests cell cycle in G0/G1 phase in WJCMSCs

**Fig. 2:**
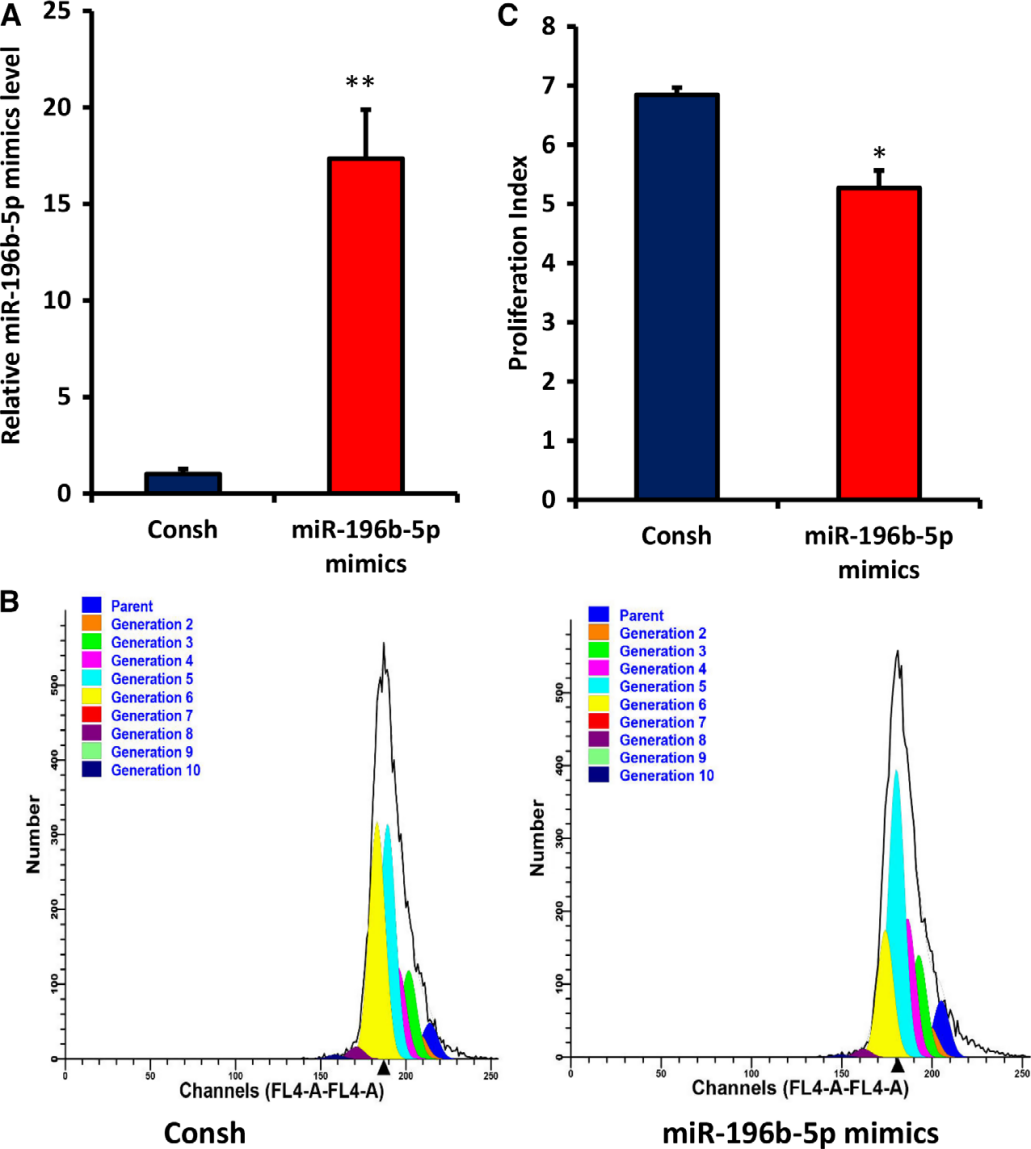
Overexpression of miR‐196b‐5p suppressed cell proliferation in WJCMSCs. (A) Real‐time RT‐PCR showed the efficiency of miR‐196b‐5p overexpression in WJCMSCs. (B, C) CFSE assay results showed that the number of cells proliferating to the sixth generation and the proliferation index in the miR‐196b‐5p mimics group were lower than those in the control group. U6 was used as an internal control for miR‐196b‐5p. Student’s *t*‐test was used to analyze the statistical significance. All error bars signify standard deviations (*n* = 3). *P ≤ 0.05, **P ≤ 0.01.

**Fig. 3:**
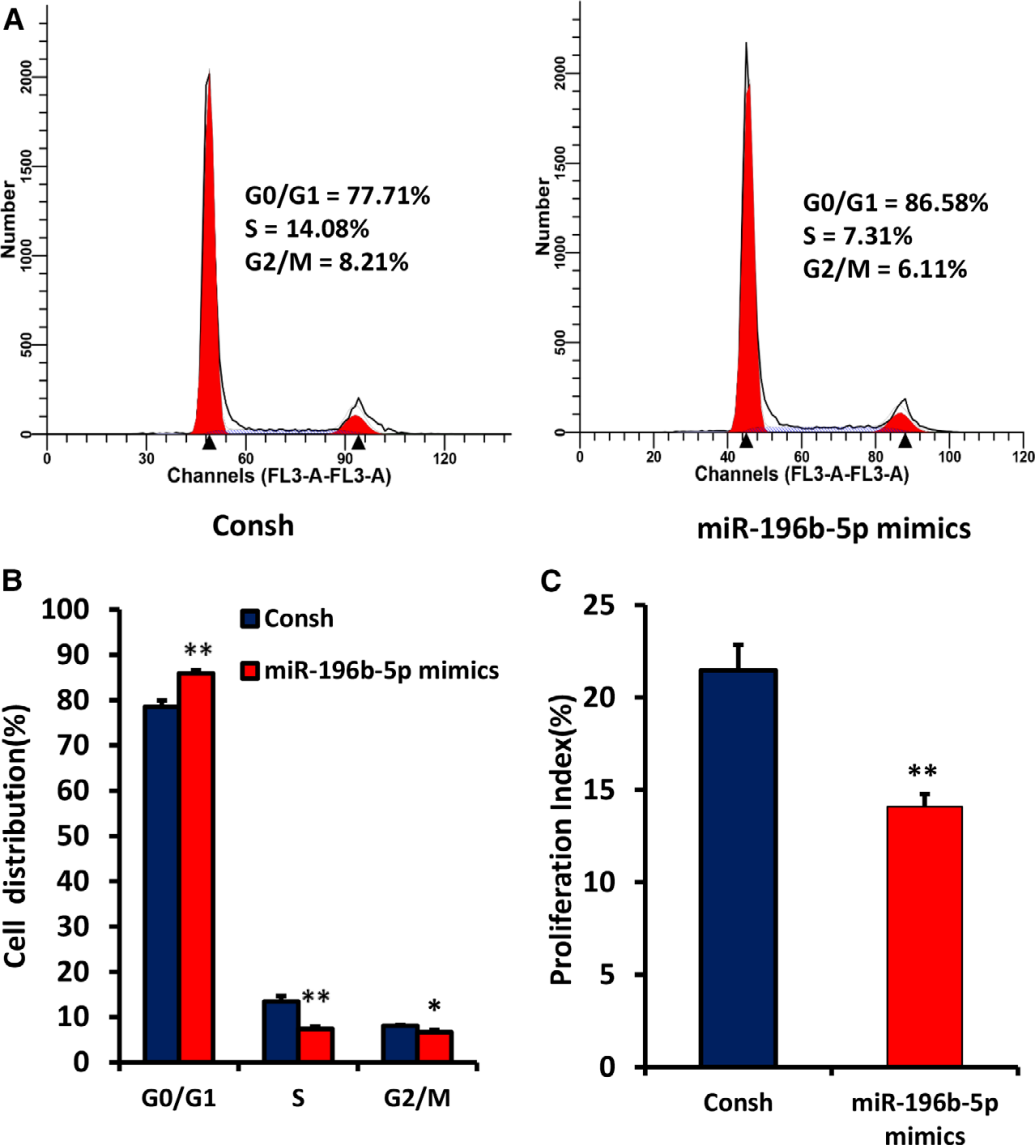
Overexpression of miR‐196b‐5p induced G0/G1 phase arrest in WJCMSCs. (A, B) The flow cytometer analysis results showed an increased cell percentage in G0/G1 phase and a decreased cell percentage in S and G2/M phase in miR‐196b‐5p‐overexpressed WJCMSCs. (C) The cell‐cycle proliferation index was calculated based on flow cytometer results. Student’s *t*‐test was used to analyze the statistical significance. All error bars signify standard deviations (*n* = 3). *P ≤ 0.05, **P ≤ 0.01.

To explore the role of miR‐196b‐5p on WJCMSC proliferation, we infected the cells with miR‐196‐5p mimics. After 3 days of 1 μg·mL^−1^ puromycin treatment, overexpression of miR‐196‐5p in WJCMSCs was confirmed by qRT‐PCR (Fig. 2A). Then, we conducted CFSE assays, and the results showed that the number of cells proliferating to the sixth generation (Fig. 2B) and the proliferation index (Fig. 2C) in the miR‐196b‐5p mimics group were significantly reduced compared with the control group. The results indicated that miR‐196‐5p mimics inhibited cell growth of WJCMSCs. Furthermore, cell‐cycle assays were performed to confirm whether the effect of miR‐196‐5p on WJCMSC growth was associated with cell‐cycle distribution. Compared with the control group, the number of cells in the miR‐196b‐5p‐overexpressed group increased significantly in G0/G1 stage but decreased significantly in S and G2/M stages (Fig. 3A,B). Moreover, cell‐cycle proliferation index detection results also showed that miR‐196b‐5p mimics suppressed cell proliferation of WJCMSCs (Fig. 3C).

### Knockdown of miR‐196b‐5p promotes cell proliferation and accelerates cell‐cycle progression in WJCMSCs

**Fig. 4:**
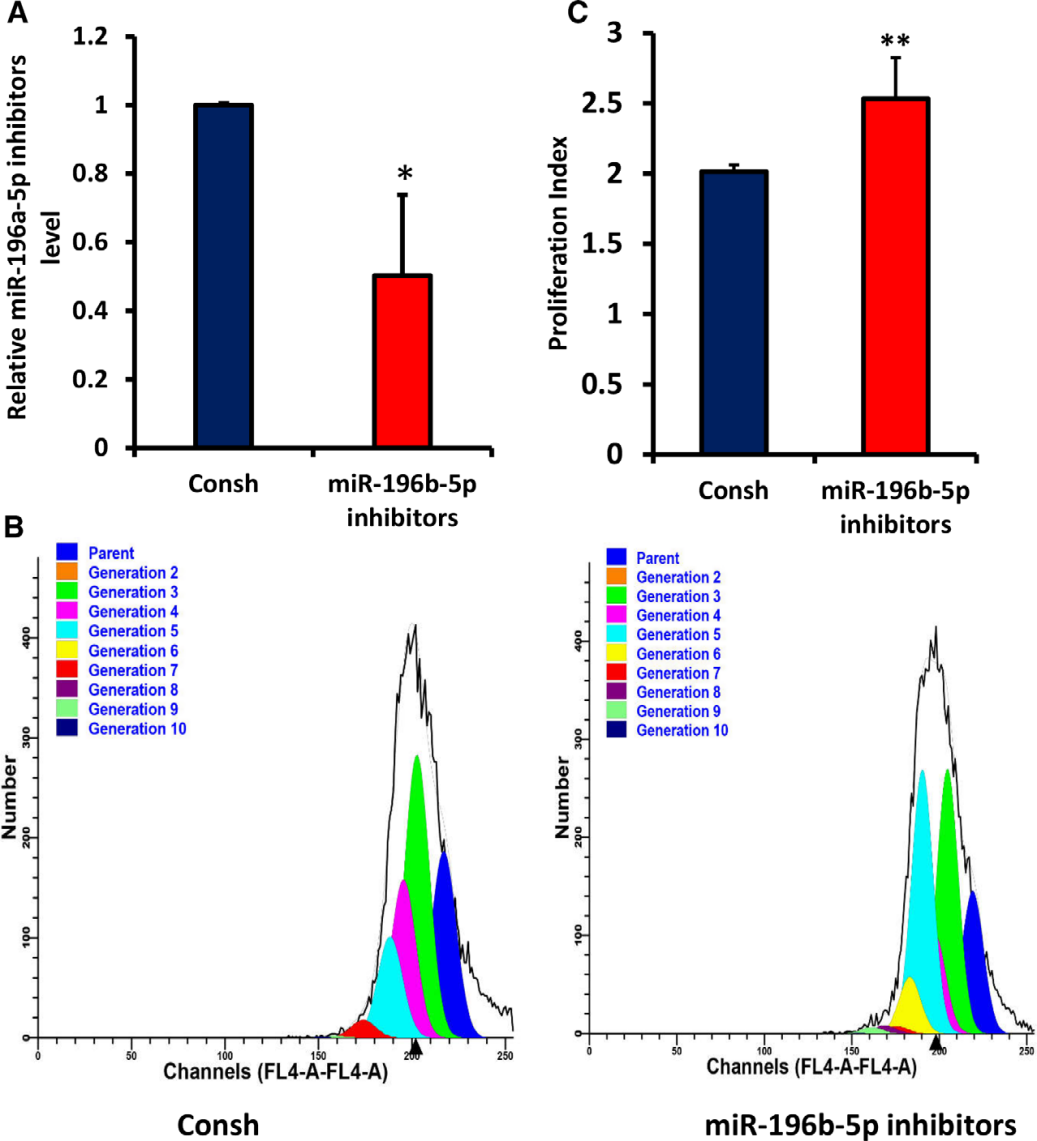
Knockdown of miR‐196b‐5p promoted cell proliferation in WJCMSCs. (A) Real‐time RT‐PCR showed the knockdown efficiency of miR‐196b‐5p in WJCMSCs. (B, C) CFSE assay results showed that the number of cells with higher proliferating algebra and the proliferation index in the miR‐196b‐5p inhibitors group were higher than those in the control group. U6 was used as an internal control for miR‐196b‐5p. Student’s *t*‐test was used to analyze the statistical significance. All error bars signify standard deviations (*n* = 3). *P ≤ 0.05, **P ≤ 0.01.

**Fig. 5:**
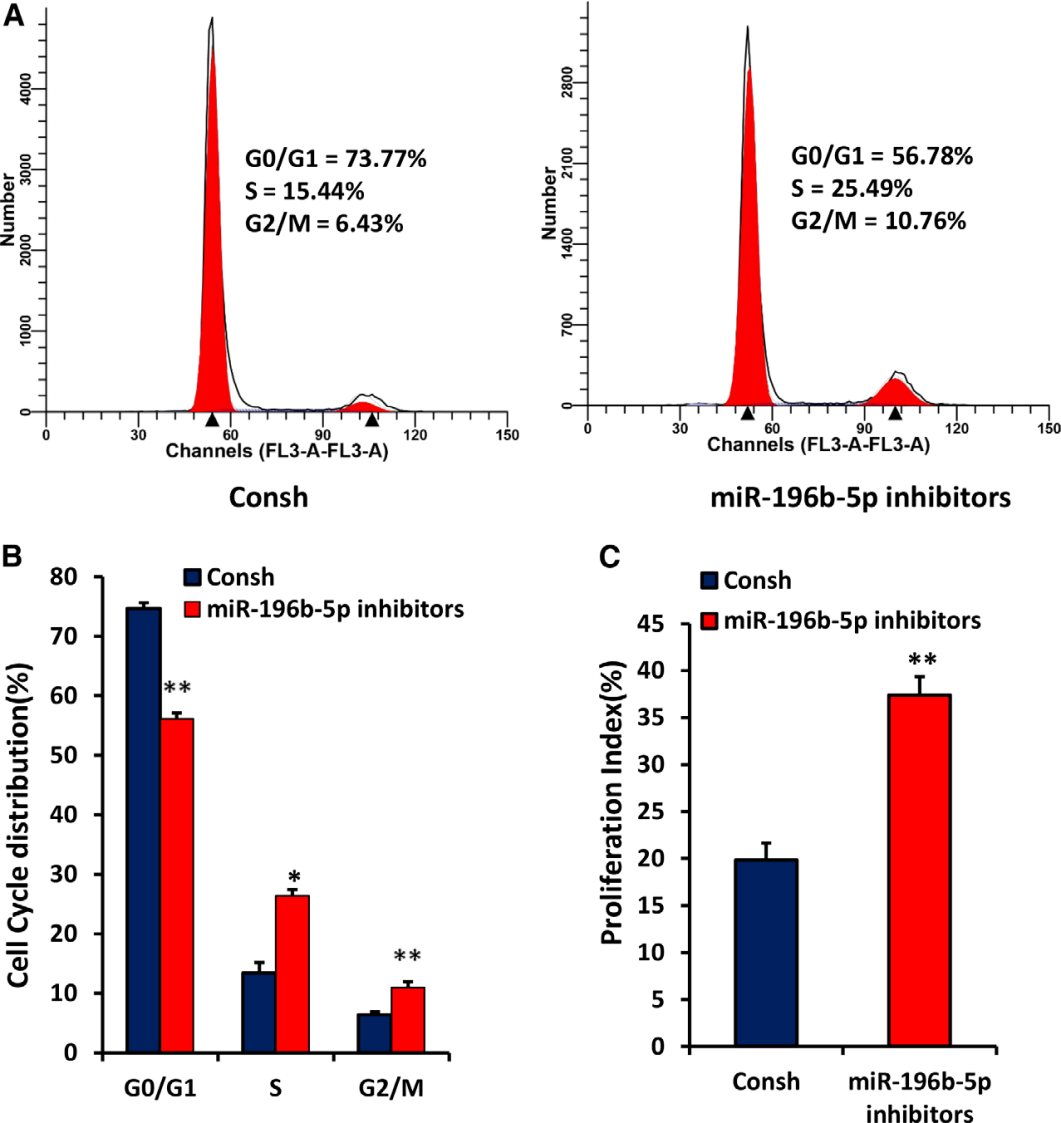
Knockdown of miR‐196b‐5p accelerated cell‐cycle progress in WJCMSCs. (A, B) The flow cytometer analysis results showed a decreased cell percentage in G0/G1 phase and an increased cell percentage in S and G2/M phases in miR‐196b‐5p knockdown WJCMSCs. (C) The cell‐cycle proliferation index was calculated based on flow cytometer results. Student’s *t*‐test was used to analyze the statistical significance. All error bars signify standard deviations (*n* = 3). *P ≤ 0.05, **P ≤ 0.01.

To further explore the role of miR‐196b‐5p on WJCMSC proliferation, we transfected miR‐196b‐5p inhibitors and Consh into WJCMSCs. After 3 days of 1 μg·mL^−1^ puromycin treatment, the knockdown efficiency of miR‐196b‐5p in WJCMSCs was detected by qRT‐PCR (Fig. 4A). CFSE assays showed that the number of cells with higher proliferating algebra (Fig. 4B) and the proliferation index (Fig. 4C) in the miR‐196b‐5p inhibitors group were significantly increased compared with the control group. The results indicated that miR‐196b‐5p inhibitors increased cell growth. Cell‐cycle assays revealed that compared with the control group, miR‐196b‐5p inhibitors significantly attenuated the G0/G1 phase ratio of WJCMSCs but significantly augmented the S and G2/M phase ratio (Fig. 5A,B). Then the cell‐cycle proliferation index further confirmed that miR‐196b‐5p inhibitors promoted WJCMSC proliferation (Fig. 5C).

### miR‐196b‐5p regulates expression of cell‐cycle‐related proteins in WJCMSCs

**Fig. 6:**
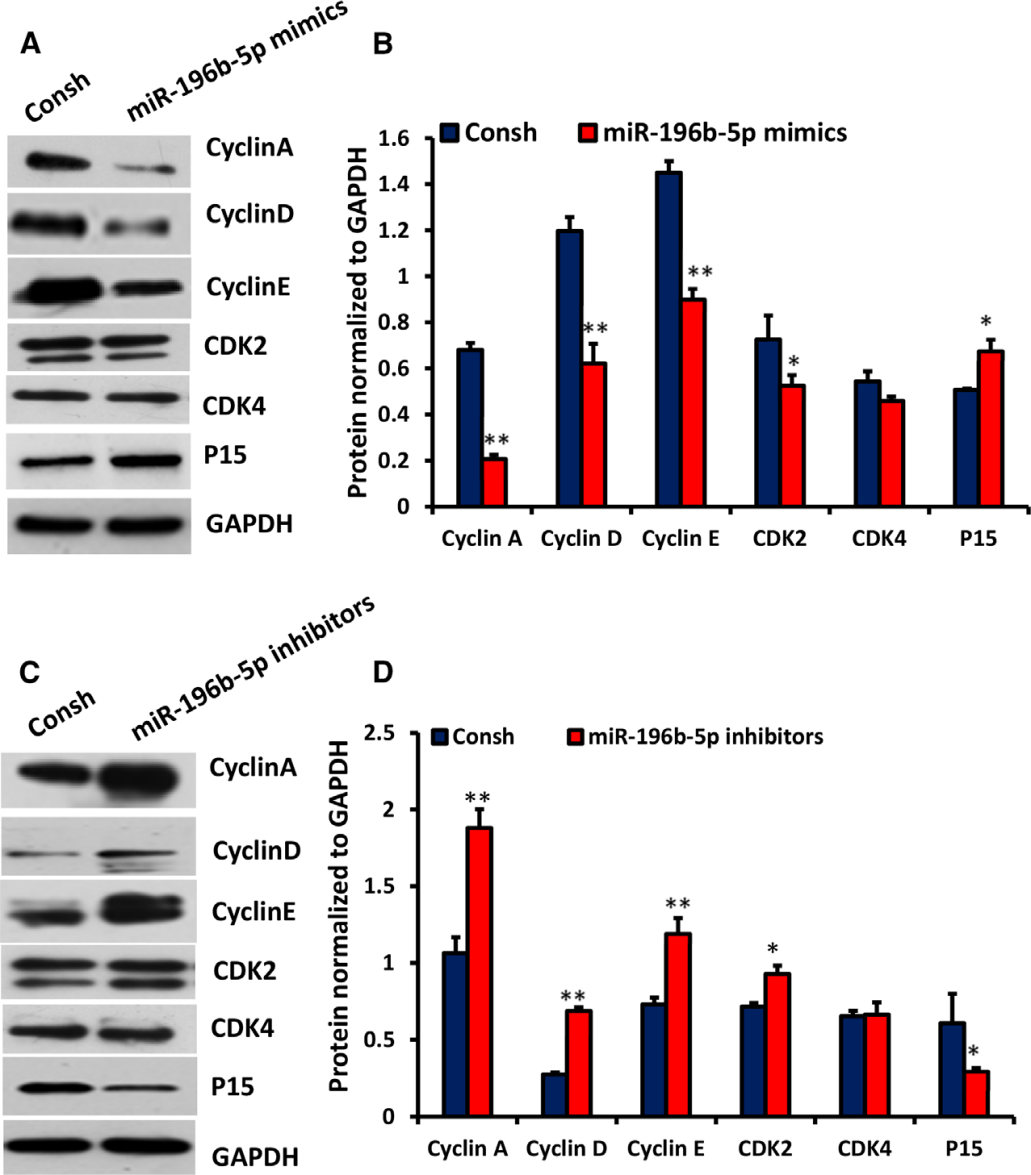
miR‐196b‐5p regulated the expressions of Cyclin A, Cyclin D, Cyclin E, CDK2 and p15^INK4b^ in WJCMSCs. (A, B) Western blot and quantitative analysis results showed the expressions of Cyclin A, Cyclin D, Cyclin E, CDK2, CDK4 and p15^INK4b^ in miR‐196b‐5p‐overexpressed WJCMSCs. (C, D) Western blot and quantitative analysis results showed the expressions of Cyclin A, Cyclin D, Cyclin E, CDK2, CDK4 and p15^INK4b^ in miR‐196b‐5p knockdown WJCMSCs. GAPDH was used as an internal control for western blot and quantitative analysis. Student's *t*‐test was used to analyze the statistical significance. All error bars signify standard deviations (*n* = 3). *P ≤ 0.05, **P ≤ 0.01.

Next, to determine the mechanism of miR‐196b‐5p on cell cycle, we measured the protein expression level of cell‐cycle‐related regulators by western blot and quantitative analysis. Under miR‐196b‐5p overexpression, the protein levels of Cyclin A, Cyclin D, Cyclin E and CDK2 decreased significantly, whereas p15^INK4b^ level increased significantly in comparison with the control group (Fig. 6A,B). Furthermore, miR‐196b‐5p knockdown up‐regulated significantly the expression of Cyclin A, Cyclin D, Cyclin E and CDK2 and down‐regulated significantly the expression of p15^INK4b^ compared with the control group (Fig. 6C,D). However, there was no significant difference in CDK4 level between miR‐196b‐5p mimics, miR‐196b‐5p inhibitors and the control groups (Fig. 6A–D).

### Sequential window acquisition of all theoretical mass spectra results analysis

**Fig. 7:**
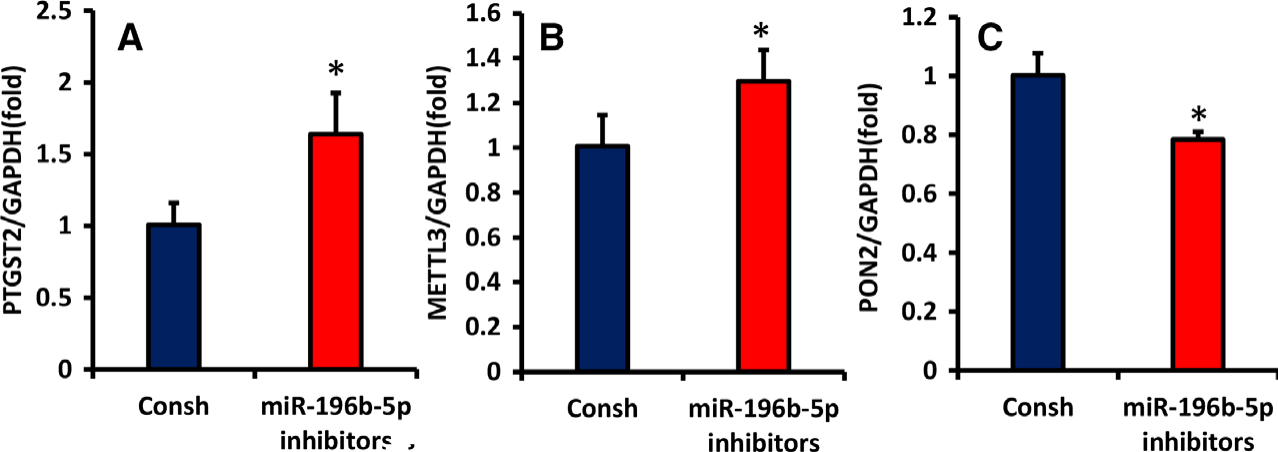
The mRNA levels of differentially expressed proteins in WJCMSCs induced by miR‐196‐5p. (A–C) Real‐time RT‐PCR analyzed the mRNA level of PTGS2, METTL3 and PON2 in miR‐196b‐5p inhibitors and the control group. *GAPDH* was used as an internal control for real‐time RT‐PCR. Student's *t*‐test was used to analyze the statistical significance. All error bars signify standard deviations (*n* = 3). *P ≤ 0.05.

To further elucidate the mechanism of miR‐196‐5p in WJCMSCs, we used sequential window acquisition of all theoretical mass spectra (SWATH‐MS) to identify differentially expressed proteins induced by miR‐196‐5p. A total of 163 proteins were found to be significantly differentially expressed between miR‐196‐5p inhibitors and the control groups, including 56 up‐regulated and 107 down‐regulated proteins (Table S1). PTGS2 and METTL3 were found to be up‐regulated, whereas PON2 was down‐regulated. We speculate that these proteins may be the direct or indirect targets of miR‐196b‐5p, regulating the proliferation of WJCMSCs. Then we detected the mRNA level of *PTGS2*, *METTL3* and *PON2* in miR‐196b‐5p inhibitors and the control group. In the miR‐196b‐5p inhibitors group, the mRNA levels of *PTGS2* and *METTL3* were significantly increased (Fig. 7A,B), whereas the mRNA level of *PON2* was significantly decreased (Fig. 7C). These results were consistent with the results of SWATH‐MS and confirmed the reliability of the SWATH‐MS data.

## Discussion

WJCMSCs are under investigation in various clinical treatment trials for regenerative medicine due to their characteristics, such as fast proliferation, rapid self‐renewal, good DT stability, low immunogenicity and feasible harvest process. Preclinical studies have shown that the therapeutic mechanisms of WJCMSCs mainly include paracrine, cell substitution and cell contact, among which paracrine is recognized as the primary mechanism [30, 31]. The basic requirement of this mechanism for clinical application is to ensure sufficient quantity and quality of cells. It has been widely recognized that the therapeutic effect of WJCMSCs will be enhanced when improving the subculture efficiency. However, studies have shown that the phenotype of WJCMSCs may be affected by culture conditions and continuous passage when the cells are expanded *in vitro* before transplantation [32]. Therefore, it is necessary to develop methods to enhance the proliferative ability of WJCMSCs to promote their clinic application.

In this study, we found that the expression of miR‐196b‐5p was significantly increased in senescent WJCMSCs, suggesting that miR‐196b‐5p may be associated with the decline of proliferation ability in aged cells. Indeed, we found that overexpressed miR‐196b‐5p inhibited WJCMSC proliferation and reduced cells of S and G2/M phases through blocking cells in G0/G1 phase, whereas miR‐196b‐5p knockdown promoted WJCMSC growth by accelerating cell‐cycle progress into S and G2/M phases. Cell proliferation is strictly controlled by cell cycle, which involves a series of complex cascade events [33]. During G1/S transition, cells are blocked in the G0/G1 phase, which means a prolonged initiation time for DNA synthesis. However, the increase of G2/M phase indicates accelerated cell mitosis and cell proliferation [34]. Our study suggested that miR‐196b‐5p might play an important role in regulating cell cycle and cell proliferation of WJCMSCs, and it may be a potential therapeutic target for improving subculture efficiency of WJCMSCs. However, the underlying mechanism is still unclear and needs further study.

The important mechanism of cell growth is mainly regulated by cell‐cycle regulatory proteins, including cyclins, CDKs and CDK inhibitors. CDK4/6 and CDK2 are activated by Cyclin D binding to CDK4/6 or Cyclin E to CDK2, but are inactive without their homologous cyclin partners [35]. In cell‐cycle regulation, the key in G1 phase is the binding of Cyclin D and CDK4/6, which drives the start of the cell cycle [36]. Cyclin E is essential for the control of the cell cycle at the G1/S transition and combines to CDK2 to make the cell cycle enter into S phase from the late G1 phase [37]. Cyclin A is induced at the G1/S boundary and binds to CDK2 in S phase and participates in the progress of S phase [38]. p15^INK4B^ is a member of the CDK inhibitor family, which can delay the progress of the G0/G1 phase through inhibiting the binding of Cyclin D and CDK4/CDK6 [39]. Our study showed that overexpression of miR‐196b‐5p down‐regulated Cyclin A, Cyclin D, Cyclin E and CDK2 and up‐regulated p15^INK4b^, whereas knockdown of miR‐196b‐5p up‐regulated Cyclin A, Cyclin D, Cyclin E and CDK2 and down‐regulated p15^INK4b^, which is consistent with the results of Li *et al*. [40]. We speculate that miR‐196b‐5p may attenuate the G1/S transition through regulating the combination of Cyclin A and Cyclin E to CDK2, and Cyclin D and p15^INK4b^ may be involved. In brief, our results showed that miR‐196b‐5p may block G0/G1 phase by down‐regulating Cyclin A, Cyclin D, Cyclin E and CDK2 and up‐regulating p15^INK4b^, which needs to be further verified.

Many studies show that miRNAs act directly or indirectly on transcripts encoding proteins related to cell proliferation and cell cycle [18]. To find the target of miR‐196b‐5p and further explain its molecular mechanism on WJCMSC proliferation, we conducted SWATH‐MS analysis in miR‐196‐5p inhibitors and the control groups. The result suggests that several differential proteins are related to cell proliferation or cell cycle, among which the expressions of PTGS2 and METTL3 were up‐regulated, whereas the expression of PON2 was down‐regulated. It was revealed that knockdown of PTGS2 (also known as cyclooxygenase‐2) could increase cells in G0/G1 phase and decrease cells in S phase, thus inhibiting the proliferation and growth of Capan‐2 cells [41]. METTL3 promoted tumor growth in bladder cancer via modulating pri‐miR221/222 maturation by an m6A‐dependent manner [42]. PON2 was involved in the regulation of C12‐HSL on the mitochondrial energy production and function of LS174T cells and the inhibition of cell proliferation [43]. Then we used qRT‐PCR to detect the mRNA levels of *PTGS2*, *METTL3* and *PON2* in miR‐196b‐5p inhibitors and the control group. The results showed that the expression levels of the selected mRNAs were consistent with the SWATH‐MS results. We speculate that *PTGS2*, *METTL3* and *PON2* might be the direct or indirect targets of miR‐196b‐5p in regulating the proliferation of WJCMSCs, which needs further exploration.

## Conclusions

This study indicated that miR‐196b‐5p suppressed cell growth by blocking the cell cycle of WJCMSCs in G0/G1 phase through down‐regulating Cyclin A, Cyclin D, Cyclin E and CDK2 and up‐regulating p15^INK4b^. This study contributed to reveal a novel molecular mechanism on WJCMSC proliferation and laid a foundation for better use of WJCMSCs in future clinical applications.

## Conflict of interest

The authors declare no conflict of interest.

## Author contributions

XH was responsible for collection and assembly of data, data analysis and interpretation, manuscript writing and final approval of the manuscript. HY, HL, YC and CZ contributed to data collection. RS and ZF were responsible for conception and design, manuscript writing and revising, financial support and final approval of the manuscript. All authors have read and approved the final version of the manuscript.

## Supporting information


**Fig. S1.** The expression of miR‐196b‐5p in WJCMSCs, ADSCs, BMMSCs, SCAPs, DPSCs, and PDLSCs. QRT‐PCR showed that the expression of miR‐196b‐5p in WJCMSCs, ADSCs, and BMMSCs increased significantly compared with SCAPs, DPSCs, and PDLSCs. U6 was used as an internal control for miR‐196b‐5p. One‐way ANOVA was used to analyze statistical significance. All error bars signify standard deviations (*n* = 3). **P ≤ 0.01.

**Table S1.** SWATH‐MS in miR‐196b‐5p inhibitors WJCMSCs.

## Data Availability

Research data are not shared. The raw data will be made available from the corresponding author upon reasonable request.
