# Supplementary figures and images for: miR‐196b‐5p inhibits proliferation of Wharton's jelly umbilical cord stem cells
Source: FEBS Open Bio. 2020 Dec 8;11(1):278–88. doi: 10.1002/2211-5463.13043 (PMC7780118; doi:10.1002/2211-5463.13043)

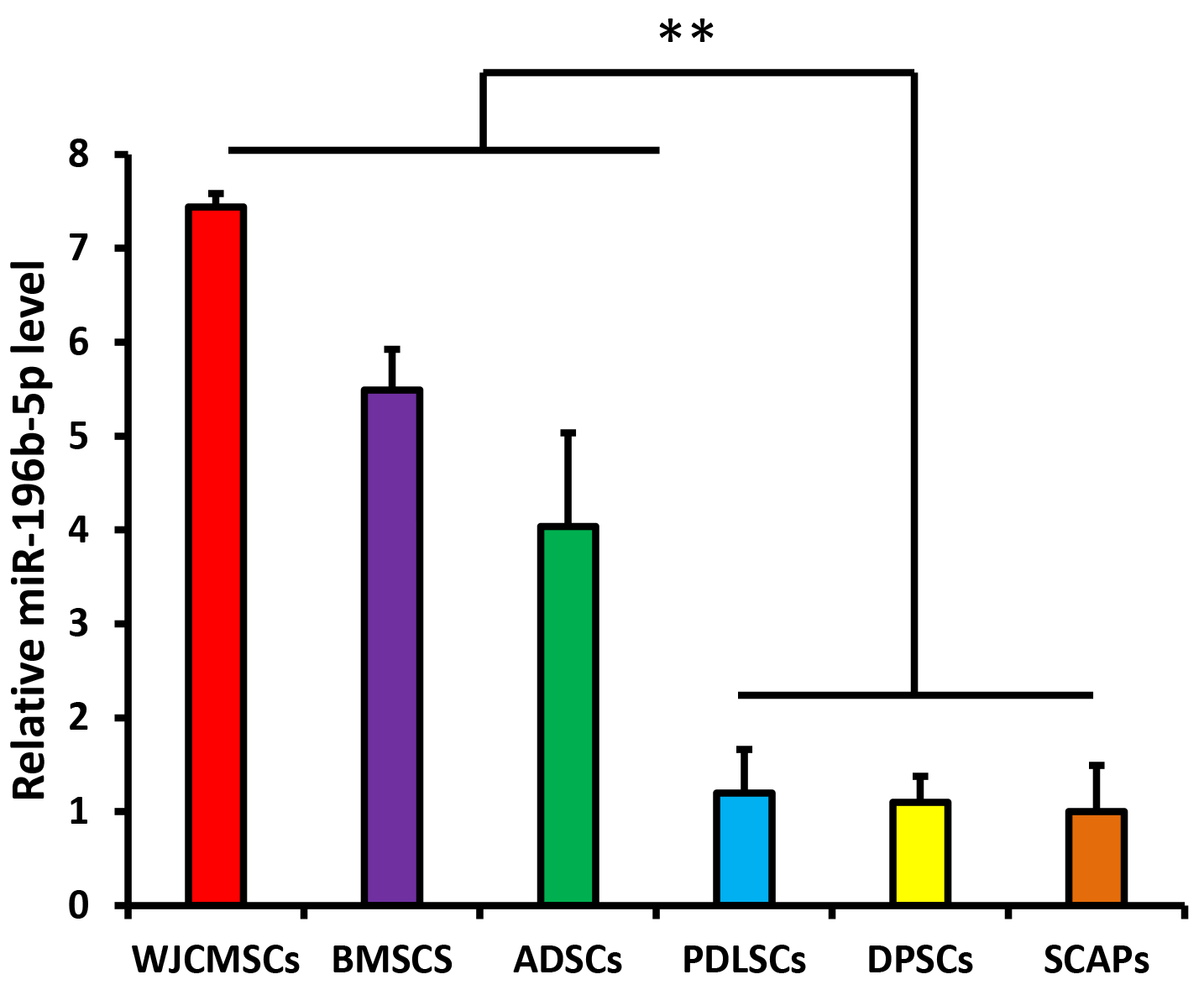

Supplement: Supplementary file 1 — Fig. S1. The expression of miR‐196b‐5p in WJCMSCs, ADSCs, BMMSCs, SCAPs, DPSCs, and PDLSCs. QRT‐PCR showed that the expression of miR‐196b‐5p in WJCMSCs, ADSCs, and BMMSCs increased significantly compared with SCAPs, DPSCs, and PDLSCs. U6 was used as an internal control for miR‐196b‐5p. One‐way ANOVA was used to analyze statistical significance. All error bars signify standard deviations (n = 3). **P ≤ 0.01. [file FEB4-11-278-s001.tif]
